# High interleukin-35 expression is associated with the severity of rheumatic mitral stenosis

**DOI:** 10.3389/fimmu.2025.1537497

**Published:** 2025-04-08

**Authors:** Ping Wang, Yaxiong Li, Li Zhao, Bin Liu, Zhibin Cai, Peng Zhang, Peng Li, Xuezhen Gao, Yong Zhan

**Affiliations:** ^1^ Department of Cardiovascular Surgery, Fuwai Yunnan Cardiovascular Hospital, Affiliated Cardiovascular Hospital of Kunming Medical University, Kunming, Yunnan, China; ^2^ Department of Cardiovascular Surgery, Yan’an Hospital Affiliated to Kunming Medical University, Kunming, Yunnan, China; ^3^ Key Laboratory of Cardiovascular Research of Yunnan Province, Yan’an Hospital Affiliated to Kunming Medical University, Kunming, Yunnan, China; ^4^ Health Examination Center, Yan’an Hospital Affiliated to Kunming Medical University, Kunming, Yunnan, China; ^5^ Division of Cardiac Surgery, Cardiovascular Center, Tufts Medical Center, Tufts University School of Medicine, Boston, MA, United States

**Keywords:** cytokine, interleukin-35 (IL-35), p35, EBI3, rheumatic mitral stenosis

## Abstract

**Background:**

Rheumatic mitral stenosis (RMS) is the most common manifestation of rheumatic heart disease, with high morbidity and mortality. Interleukin-35 (IL-35) is a novel anti-inflammatory cytokine associated with many autoimmune diseases. However, the relation between IL-35 expression and RMS remains unknown. We aimed to study IL-35 expression in RMS and its association with disease progression.

**Methods:**

IL-35 concentration was analyzed in blood samples from 40 patients, including 20 moderate, 20 severe RMS, and 20 healthy controls by ELISA. Mitral valve (MV) IL-35 expression was determined by western blot and immunohistochemistry in patients with RMS (22 and 29 cases, respectively) in comparison to control specimens with mitral valve prolapsed (5 cases, respectively).

**Results:**

IL-35 levels were significantly elevated in the blood of the RMS patients compared to those from healthy subjects(p<0.05) and positively correlated with the severity of RMS (r=0.317, p<0.05). The expression of IL-35 and its subunits (p35 and EBI3) was also detected in MV tissues of patients with moderate or severe RMS. The expression of IL-35 and its subunits (p35 and EBI3) had a positive association with the severity of RMS in MV tissues (r=0.528, p<0.01; r=0.561, p<0.001; r=0.456, p<0.01). Co-localization of p35 and EBI3 was seen in MV tissues of RMS patients in a predominantly perivascular pattern.

**Conclusion:**

We show for the first time an increase of IL-35 level in the blood and MV tissues of RMS patients, which is strongly correlated with the severity of RMS. These results suggest that IL-35 plays an important regulatory role in the progression of RMS.

## 1 Introduction

Rheumatic heart disease (RHD) is an autoimmune disease resulting from acute rheumatic fever (ARF) (1, 2). Although many measures, including prompt treatment of streptococcal pharyngitis and improvement of social health systems, have been implemented to prevent RHD, it is still a common cardiovascular disease with high morbidity and mortality, especially in developing countries (3–6). The disease causes more than 300,000 deaths annually worldwide (3). Rheumatic mitral stenosis (RMS) is the most common manifestation of RHD, though other heart valves may also be involved (6, 7).

The etiology of RHD remains unclear (7, 8). Studies have shown that cytokines play major roles in the susceptibility and progression of RHD (8). Various pro-inflammatory cytokines, including tumor necrosis factor (TNF)-α and interleukin (IL)-1, IL-2, IL-6, IL-8, and IL-23 or anti-inflammatory cytokines such as IL-10 have been found to increase in either ARF or RHD (8, 9). The upregulation of pro-inflammatory cytokines appears universal and extensive in this setting. In contrast, few studies on pro-inflammatory cytokines and anti-inflammatory in the late stage of RHD, such as RMS, suggest immune responses in the spectrum RHD may differ. Emerging evidence suggests that T cells are involved in the pathogenesis and development of chronic RMS (10, 11).

IL-35, a new and important immunoregulatory cytokine, regulates immune reactions through its pleiotropic effects (12). As a heterodimer composed of p35 and EBI3 subunits and secreted mainly by Treg cells (Tregs) and Breg cells (Bregs) (7, 13), it induces the proliferation of Breg and Treg cells, expands IL-10 secretion, and suppresses T effector cell proliferation and Th17 cell development (14–16). These suggest a potential role of IL-35 in the development of RMS. We aimed to determine IL-35 protein expression in RMS patients and the correlation between IL-35 and the severity of mitral valve stenosis.

## 2 Methods

### 2.1 Study subjects

Ninety-one patients with RMS were enrolled in Cardiovascular Center at Kunming Medical University-affiliated Yan’an Hospital and Yunnan Fuwai Hospital from September 2019 to December 2022. Among them, 23 were males, and 68 were females. During the same period, 10 patients (3 male and 7 female) with mitral valve prolapse (MVP) who received mitral valve replacement in the two hospitals and 20 healthy people (7 male and 13 female, mean age 42.1 ± 1.9 years) from the Health Examination Center of Yan’an Hospital were also enrolled in the study as controls (**Tables 1**–**3**).

**Table 1:** Clinical characteristics of RMS patients and healthy controls for ELISA.

	RMS patients		Controls (n=20)	P
	Moderate (n=20)	Severe (n=20)		
Gender (male/female)	3/17	4/16	7/13	0.298
Age (years)	47.65±7.4714	48.00±7.19	39.45±7.149	0.125
LVEF (%)	55.00±5.49	53.75±5.06	—	0.458

Data are ratio or mean ± standard deviation.

ELISA, enzyme-linked immunosoebent assay; LVEF, left ventricular ejection fraction; RMS, rheumatic mitral stenosis.

**Table 2:** Clinical characteristics of RMS patients and controls for Western blot.

	RMS patients		Controls (n=5)	P
	Moderate (n=10)	Severe (n=12)		
Gender (male/female)	1/9	4/8	1/4	0.420
Age (years)	42.60±9.47	45.42±7.549	46.20±11.71	0.697
LVEF (%)	56.80±7.63	57.75±10.16	46.60±7.06	0.105

Data are ratio or mean ± standard deviation.

LVEF, left ventricular ejection fraction; RMS, rheumatic mitral stenosis.

**Table 3:** Clinical characteristics of RMS patients and controls for immunohistochemistry.

	RMS patients		Controls (n=5)	P
	Moderate (n=14)	Severe (n=15)		
Gender (male/female)	6/8	5/10	2/3	0.399
Age (years)	47.00±7.168	46.80±7.830	46.60±3.435	0.993
LVEF (%)	58.50±7.823	57.87±4.838	53.00±7.071	0.269

Data are ratio or mean ± standard deviation.

LVEF, left ventricular ejection fraction; RMS, rheumatic mitral stenosis.

Inclusion criteria for RMS patients were the presence of RHD, diagnosed, and classified by color Doppler echocardiography according to World Heart Federation criteria for echocardiographic diagnosis of RHD in 2012 (17). MV stenosis in this study is classified as moderate and severe according to mitral valve area (MVA): MVA ≤ 1.5 cm^2^ is considered moderate MS, MVA ≤ 1.0 cm^2^ is considered severe MS (18). Trans-thoracic echocardiography was performed in all subjects (EPIQ 7C; Philips, USA; with 1 to 5 MHz transducer). The criteria for the control group of patients with MVP included congenital leaflet prolapse and/or chordal elongation/agenesis/traumatic rupture, visualized by echocardiography (19) and surgical procedures. Exclusion criteria for all enrolled subjects were as follows: systemic autoimmune diseases, allergy, immunodeficiency, immune response modifying drugs, monoclonal antibodies, acute or chronic infectious diseases, ischemic heart disease, hypertension, diabetes, rheumatic mitral and aortic valves disease, atrial fibrillation, thrombosis, pregnancy, pulmonary, renal or hepatic diseases, and malignancies. Patients having clinical or biochemical evidence of rheumatic activity were also excluded. The diseases excluded from the study are likely to affect IL-35 expression based on extensive literature.

The 91 RMS subjects (involving 44 moderate and 47 severe RMS) and 30 subjects as controls were divided into three groups for three independent experiments with age and gender-matched. The baseline clinical characteristics of the subjects were also similar among the three groups of ELISA, Western blot and Immunohistochemistry (**Tables 1**–**3**). A written informed consent was signed by each subject participating in the study, and all study protocols were approved by the Ethics Committee of Kunming Medical University and the Ethics Committee of Fuwai Yunnan Cardiovascular Hospital, with the code of ethics, IRB2021-BG-006.

### 2.2 Enzyme-linked immunosorbent assay

All blood samples were obtained from the superior central venous catheter. They were collected in ethylene diamine tetraacetic acid tubes and processed within 30 min by centrifugation at 1000 g for 15 min at 4°C. The supernatant was stored at -80°C.

Quantification of IL-35 cytokine was performed using an ELISA kit (HEC008Hu, Cloud-Clone Corp, USA) according to the manufacturer’s instructions. The optical density of each well was acquired at 450 nm with the equipment (Varioskan LUX, Thermo, Finland). Each test was conducted in duplicate for each sample. IL-35 concentrations from each sample were estimated from the standard curve using SKANIT analysis software (v. 4.1, Thermo, Vantaa, Finland).

### 2.3 Western blot

The Western blot procedure followed a protocol described by Li et al. with modifications (20). MV frozen tissue samples were grounded and lysed with radioimmunoprecipitation assay buffer (P0013, Beyotime, China) and treated with protease and phosphatase inhibitors for 30 min on ice. After using an ultrasonic cell disruptor to break all cell clusters, the samples were centrifuged at 12,000 g for 15 min at 4°C. Protein contents were quantified with Butyleyanoacrylate Protein Assay Kit (P0010, Beyotime, China). Equal amounts (30μg) of protein samples were subjected to 12% sodium dodecyl sulfate-polyacrylamide gel electrophoresis and transferred onto polyvinylidenefluoride (PVDF) membranes (ISEQ00010, Millpore, Germany). The membranes were blocked in 5% bovine serum albumin solution and incubated with primary rabbit anti-IL-35 monoclonal antibodies (mAbs) (701101, Thermo, USA), followed by incubation with goat secondary mAbs (SA00001-2, Proteintech, USA) conjugated with horseradish peroxidase. As an internal control, mouse anti-human β-actin mAb (Proteintech, USA) and peroxidase-conjugated anti-mouse IgG (H+L) (Proteintech, USA) were used. Immunoreactivity was visualized by enhanced chemiluminescence detection reagents (MA0186, Meilunbio, China). Protein bands were then analyzed using ImageJ software (v. 1.50d, NIH, USA).

### 2.4 Immunohistochemistry and immunofluorescence

Immunohistochemical staining for p35 or EBI3 was performed using the avidin-biotin peroxidase method, as previously described (21). Sections of 4 μm, which had been conventionally dewaxed with xylene and rinsed with phosphate-buffered saline (PBS) three times (3 min for each), underwent high-voltage antigen retrieval for 2.5 min. Then, they were cooled to room temperature and washed twice in PBS (0.01M pH 7.4). After immersing in 3% H_2_O_2_ for 10 min and PBS washes, the sections were permeated with 0.3% Triton X-100, and blocked with 10% normal goat serum for 1h at room temperature. Serial valve sections were incubated with a primary rabbit anti-EBI3 mAb (1:100, ab83896, Abcam, MA, USA) or a primary rabbit anti-p35 mAb (1:100, 2680s, Cell Signaling, USA) at 4°C overnight. Following incubation with a goat anti-rabbit IgG (H+L) second Ab (1:200, 04-15-06, KPL, USA) for 45 min at 37°C, the sections were enveloped with diaminobenzidine (MB9896, Meilunbio, China) for 5 to 10 min. Thereafter, the sections were counterstained with hematoxylin for 5 to 8 min. The slides were then dehydrated in ethanol, cleared in xylene and mounted with neutral balsam. Sections incubated with PBS solution instead of primary antibodies were used as negative controls. Images were captured with a light microscope (DS-Ri1, Nikon, Japan). Each section was randomly imaged with 3 non-overlapping high-power fields to measure the areas with positive staining and the positive area ratio with NIH ImageJ software (v.1.50d, NIH, USA).

For multiplex immunofluorescence, primary antibodies were the same as the immunohistochemical staining. The sections were incubated with goat anti-rabbit IgG-H&L (Alexa Fluor^®^ 647) secondary Abs (1:200, ab15015, Abcam, MA, USA) labeled for EBI3 or goat anti-rabbit-IgG H&L (FITC) secondary Abs (1:200, ab6717, Abcam, MA, USA) labeled for p35 for 45 min at 37°C, respectively. After washing three times with PBS, the sections were incubated with 4,6- diamidino-2-phenylindole (DAPI) for 5 min at 37°C. After washing, images (1000X) were captured with Laser Scanning Confocal Microscope (LSCM) (A1, Nikon, Japan) and viewed using NIS-Elements Viewer (v. 4.20, Nikon, Japan).

### 2.5 Statistical analysis

Data were presented as mean ± standard deviation for continuous variables. Comparisons between two groups were done using unpaired *t-*test for normal-distribution data, and Mann–Whitney U-test for non-normal distribution data. Chi-square test was used for categorical variables. One-way analysis of variance with Turkey Kramer multiple comparison test was used to compare multiple groups. Spearman’s correlation was used to calculate correlations of parameters. The sample sizes for ELISA,Western blot and immunohistochemistry experiments, were all calculated to the minimum required using a statistics formula ( $n=\frac{{\left(Z_{a}+Z_{\beta }\right)}^{2}\times 2\times \sigma^{2}}{\delta^{2}}$ ). In all analysis, a two- tailed value of P < 0.05 was considered statistically significant. Statistical analyses were performed using GraphPad Prism (v. 8.0, GraphPad Software, USA).

## 3 Results

### 3.1 Elevated IL-35 in the plasma of RMS patients and its correlation with the severity of RMS

**Figure 1:**
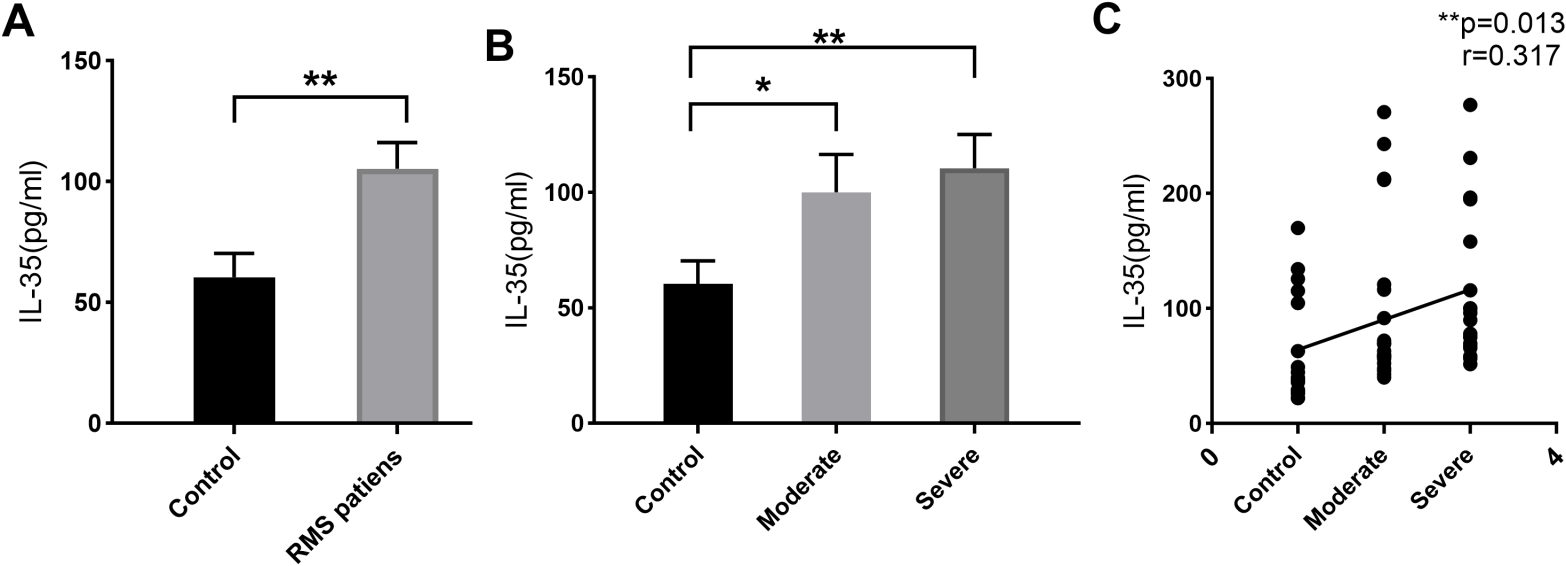
IL-35 expression in the plasma of RMS patients and controls by ELISA. **(A)** Elevated plasma IL-35 level of RMS patients compared to healthy volunteers. **(B)** Comparisons of IL-35 in moderate and severe RMS patients separately to controls. **(C)** A positive correlation between plasma IL-35 level and the severity of RMS. ns, non-significant;*p <0.05 and **p <0.01.

Plasma IL-35 levels in 40 RMS patients and 20 healthy controls were detected by ELISA. Their clinical characteristics are summarized in **Table 1**. The plasma IL-35 levels in RMS patients are significantly higher than that in healthy controls (105.2 ± 68.7 pg/ml vs. 60.5 ± 43.96 pg/ml, *P* = 0.01<0.05) (**Figure 1A**). Moreover, moderate and severe RMS patients had higher plasma IL-35 concentrations than the control group, respectively (100.1 ± 73.06 pg/ml vs. 60.5 ± 43.96 pg/ml, *P*=0.044<0.05; 110.4 ± 65.47 pg/ml vs. 60.5 ± 43.96 pg/ml, *P*=0.007<0.01) (**Figure 1B**). We observed a significant positive correlation between plasma IL-35 levels and the severity of mitral stenosis in RMS patients (*P=*0.013<0.05) (**Figure 1C**).

### 3.2 IL-35 expression in impaired MV of RMS patients

**Figure 2:**
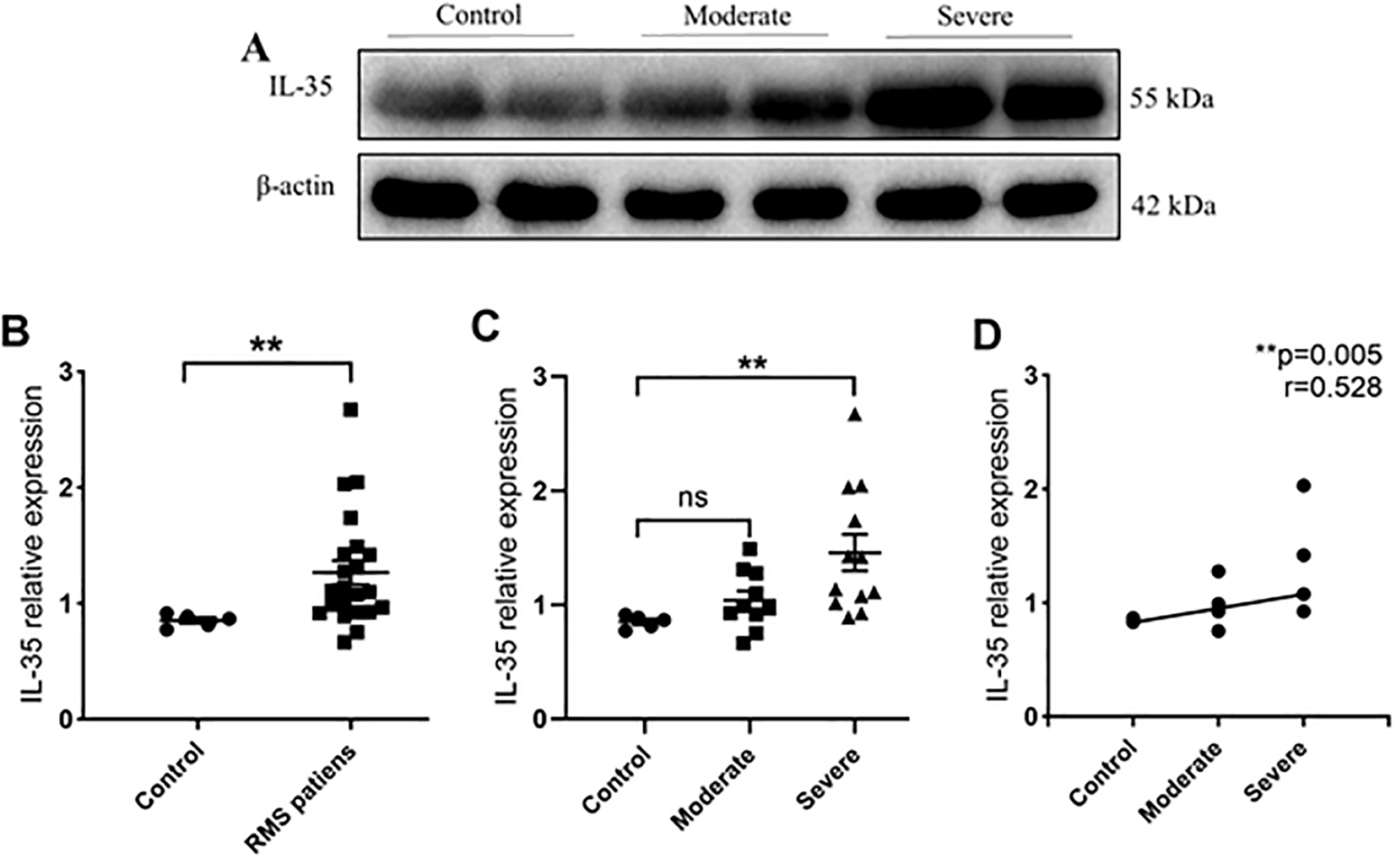
IL-35 expression in MV tissues of RMS patients and controls. **(A)** Western blot analysis of IL-35 expression in controls, moderate and severe RMS patients. β-actin was used as an internal control. **(B)** IL-35 expression in the MV tissues of RMS patients compared to controls (MVP). **(C)** Comparisons of IL-35 expressions in MV tissues of moderate and severe RMS patients vs. controls (MVP). **(D)** A positive correlation between p35 expression in MV tissue and the severity of RMS. ns, non-significant; **p < 0.01.

IL-35 expression was detected in protein extracts from the MV samples of 22 RMS (10 moderate and 12 severe) and five control (MVP) patients (**Table 2**). Western blot was performed to detect the presence of IL-35 in each sample using β-actin as the internal control (**Figure 2A**). The levels of IL-35 protein were quantitated and normalized against β-actin levels. Higher levels of IL-35 protein were detected in the RMS group compared to the control group (*P*=0.006<0.01) (**Figure 2B**). A subgroup analysis revealed that IL-35 expression was significantly higher in the MV specimens of the severe but not the moderate RMS group in comparison with the control (MVP) group (*P*=0.009<0.01) (**Figure 2C**). IL-35 levels correlated with the severity of RMS (r= 0.528, *P*=0.005 <0.01) (**Figure 2D**).

### 3.3 Localization of IL-35 protein expression in MV tissues of RMS patients by immunohistochemistry and immunofluorescence

**Figure 3:**
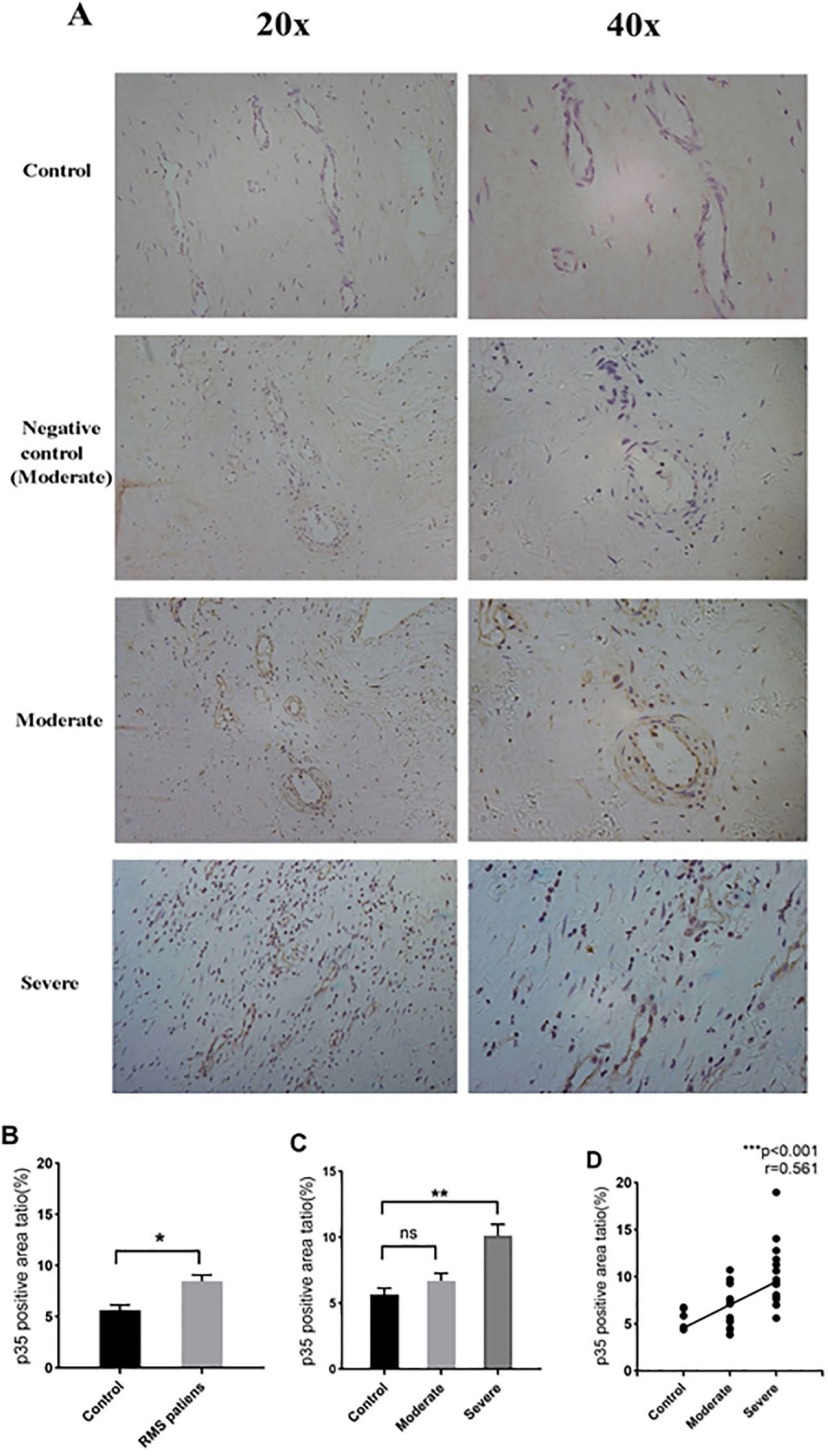
Expression and localization of p35 in MV tissues of RMS patients and controls (MVP) by immunohistochemistry. **(A)** Representative images (20×magnification and 40×magnification) of p35 expression in MV tissues (brown). **(B)** p35 expression in MV tissues of the RMS group compared to the control (MVP) group. **(C)** Quantitative analysis of p35 subunit in the control, moderate and severe RMS groups. **(D)** A positive correlation between p35 expression in MV and the severity of RMS. ns, non-significant; *p<0.05 and **p < 0.01.

**Figure 4:**
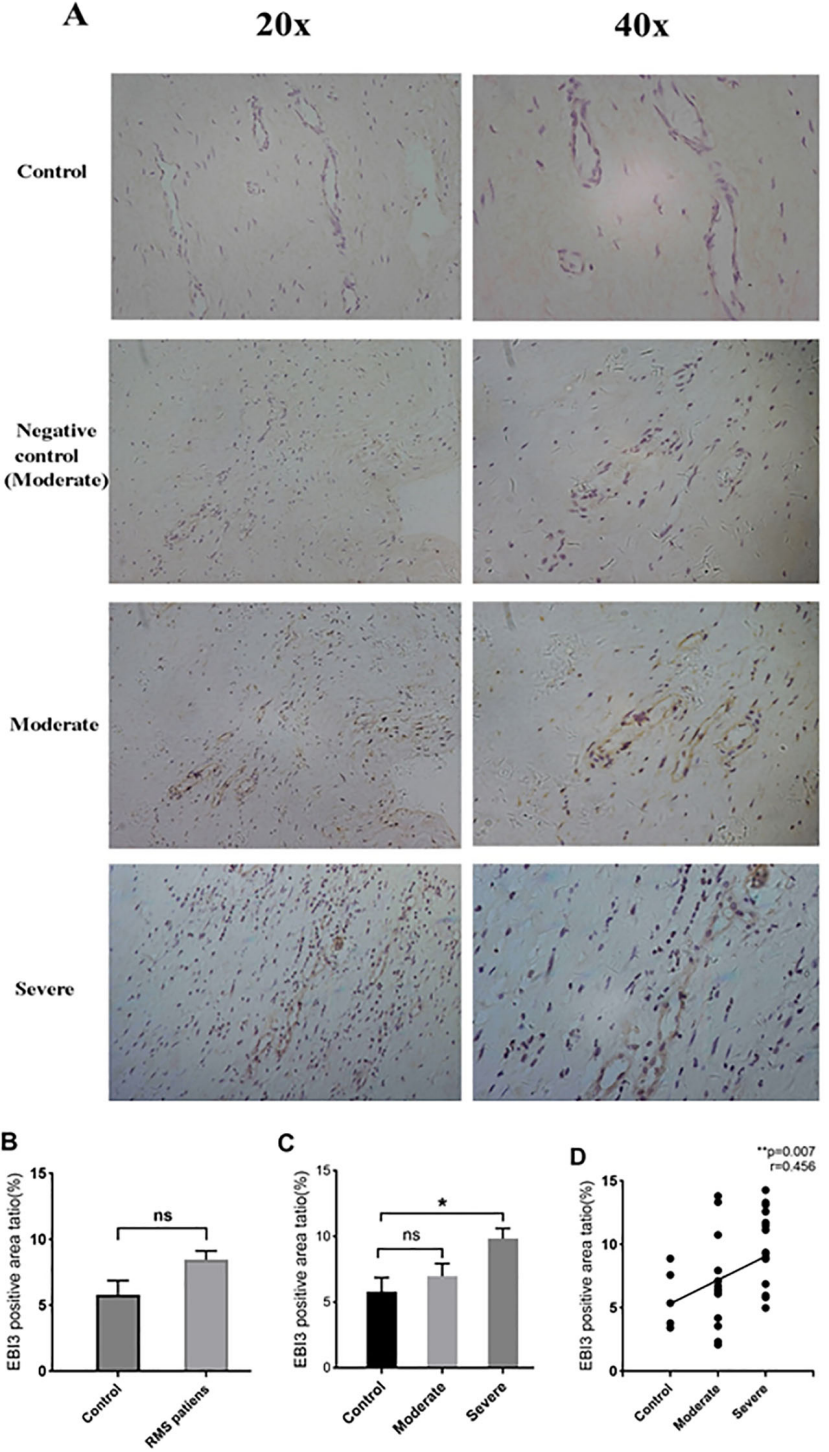
Expression and localization of EBI3 in MV tissues of RMS patients and the controls (MVP) by immunohistochemistry. **(A)** Representative images (20×magnification and 40×magnification) of the EBI3 subunit in MV tissues (brown). **(B)** EBI3 expression in MV tissues of the RMS group compared to the control (MVP) group. **(C)** Quantitative analysis of the EBI3 subunit in the control, moderate and severe RMS groups. **(D)** A positive correlation between p35 expression in MV and the severity of RMS. ns, non-significant; *p<0.05; and **p < 0.01.

IL-35 is composed of p35 and EBI3 subunits. Therefore, p35 or EBI3 expression could indirectly reflect IL-35 expression. We utilized p35 and EBI3 mAbs to study the pattern and level of IL-35 expression in the MV tissues of all three groups (**Table 3**). Adjacent sections of each specimen were stained for p35 and EBI3 in the MV tissue by immunohistochemistry. We found that p35 and EBI3 have a similar expression pattern in the MV tissues of moderate or severe RMS patients (**Figures 3A**, **4A**). The vast majority of p35 and EBI3 positive cells were located in close proximity to the vasculature, whereas the minority of them dispersed in interstitial tissues.

Quantification of the immunohistochemistry images revealed an increased p35 expression in the RMS patients vs. control group (*P*=0.034<0.05) (**Figure 3B**). The difference in p35 expression was significant between the severe RMS and control groups (*P*=0.009<0.01), but not significant between the moderate RMS and control groups (*P*=1>0.05) (**Figure 3C**). Correlation analysis revealed that p35 expression is associated with the severity of RMS (r= 0.561, *p<*0.001) (**Figure 3D**).

EBI3 expression in the RMS patients overall was also numerically higher in comparison to the control group, but the difference did not reach statistical significance (*P*=0.114 >0.05) (**Figure 4B**). The severe RMS group, but not the moderate RMS group had significant higher expression of EBI3 compared to the control group (*P*=0.041 <0.01) (**Figure 4C**). We also found that EBI3 protein expression correlates with the severity of RMS (r=0.456,*p*= 0.007<0.01) (**Figure 4D**).

**Figure 5:**
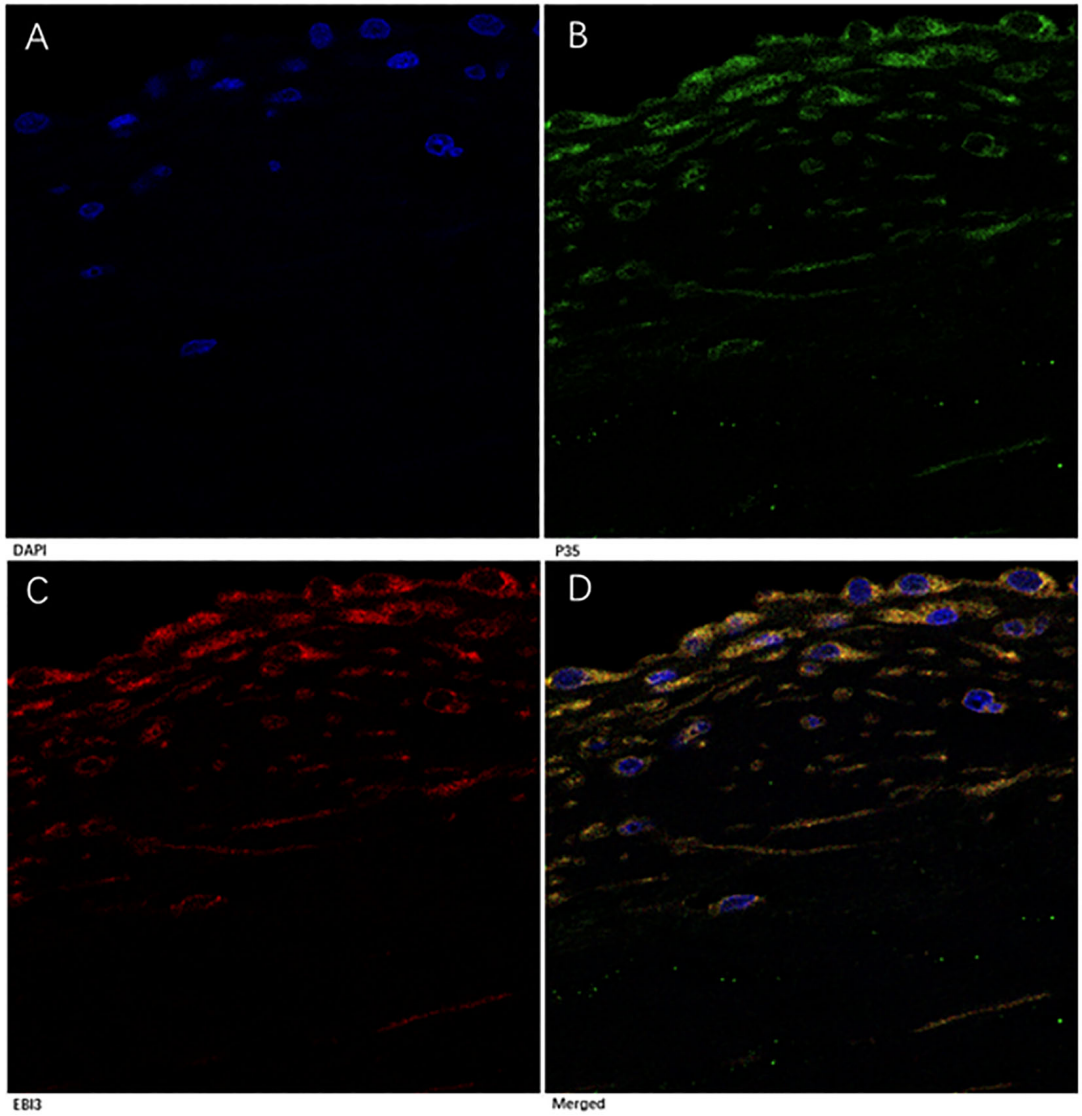
Immunofluorescence analysis of p35 and EBI3 expression in MV tissues of RMS patients. A representative severe RMS case is shown with 1000× magnifications. **(A)** DAPI staining for nucleus (blue). **(B)** p35 expression (green). **(C)** EBI3 expression (red). **(D)** Merged images of **(A-C)**, Yellow represents co-localization of p35 and EBI3 expression (depicts IL-35).

To confirm IL-35 expression in MV tissues, we performed a multi-color immunofluorescence analysis. In the MV tissues from all three groups, p35 and EBI3 expressions were well co-localized (**Figure 5**). They appeared in the perivascular region, although sparse co-expression of p35 and EBI3 was also identified in the interstitial tissue.

## 4 Discussion

In the present study, we demonstrated that RMS patients had higher plasma IL-35 concentrations than healthy individuals. Furthermore, we found that IL-35 expression in the blood and MV tissues correlated with the severity of RMS. We selected MVP patients as the control group for Western blot and immunochemistry analysis because it is difficult to acquire MV tissues from healthy people (22). Our results suggest that IL-35 might play a role in the development of RMS. The correlation between RMS and IL-35 has never been reported in the literature.

IL-35 regulates innate and adaptive immune responses, and its suppressive activities have been shown in many autoimmune and infectious diseases (23, 24). It initiates an anti-inflammatory response by inducing Treg and Breg cells (16) and blocking the accessibility of pro-inflammatory cytokines to their receptors (10). Plasma IL-35 concentration is often reduced in acute coronary syndrome, and it has been shown that IL-35 can rescue cardiac function by inhibiting ACS-induced inflammatory infiltration and promoting angiogenesis (12). However, its role in the development of RMS has not been defined. Our finding that the level of IL-35 expression in both blood and MV tissues corresponds to the progression of RMS implies that IL-35 could participate in RMS development, and its increased levels might reflect a chronic rather than an acute inflammation.

In chronic RHD, a low-grade inflammatory response can be present, as abundant immune cells are found in the deformed valvular tissue despite an absence of streptococcal antigens (1). Such an inflammatory response continues to progress locally, although no systemic manifestations are exhibited. The cytokine profiles vary in patients with RHD versus ARF, suggesting that immune responses vary in different phases during the development of RHD (1). In contrast to the universal presence of pro-inflammatory cytokines, such as TNF-α, interferon-γ, IL-6, IL-17, and IL-23 in ARF/RHD (1, 8), high levels of anti-inflammatory cytokines such as IL-10 were mainly found in patients with severe valvular diseases (25, 26). Our research revealed that Il-35 levels were elevated in severe RMS cases. We assessed this increase might result from pro-inflammatory cytokines attempting to suppress immune responses.

In contrast to our findings, previous studies showed that plasma levels of IL-35 were reduced in autoimmune diseases such as systemic sclerosis (27) and psoriasis (28). The rationale behind the reduced levels of IL-35 in autoimmune diseases is that IL-35 is an immune suppressive cytokine, and loss of immune suppression leads to the development of autoimmune diseases. However, IL-35 is known to exert its immunosuppressive effects by inducing IL-10 production (14) and inhibiting IL-17 cells from producing IL-17, a pro-inflammatory factor (16). Therefore, we propose that increased production of IL-35 may be essential for stimulating IL-10 secretion and/or reducing IL-17 production to suppress immune responses during the advanced stage of RMS.

Due to the lack of antibodies to IL-35 during our study, we performed immunohistochemistry with individual mAbs to p35 and EBI3 proteins. While p35 and EBI3 are also subunits of IL-12 and IL-27, respectively (12), there is a possibility the positive staining in the MV specimens of RMS patients could be due to cross-reactivity with other cytokines. However, no reports in the literature show either IL-12 or IL-27 participates in the development of RHD. Our observation of p35 and EBI3 co-localization in the impaired MV by immunofluorescence confirmed IL-35 expression. In addition, intracellular co-localization of p35 and EBI3 by immunofluorescent staining suggests a *de novo* production of IL-35 in the deformed MV. Increased plasma IL-35 levels were also seen in patients with advanced RMS and correlated with the severity of the disease. It remains to be determined if the high IL-35 levels in the plasma are the result of the increased IL-35 expression in the MV tissues of patients with RMS, or vice versa. Therefore, IL-35 may be considered a maker of mitral valve severity in RMS.

Compared to the pathological MV specimen from patients with MVP, the MV of RMS showed perivascular accumulation of cells expressing p35 and/or EBI3. We did not intend to verify the types of these p35 and EBI3-expressing cells. However, they are possibly lymphocytes based on their appearance, as is known that IL-35 is mainly produced by activated Tregs (15). As part of immune responses, the migration of regulatory and effector immune cells resulted in the infiltration and proliferation of these cells in tissues, triggering cytokine production and subsequent inflammatory responses (2). It is interesting that scattered p35 and EBI3 staining was also detected in the interstitial tissue, particularly in severe RMS. The locations of these cells centered around vasculature and in the interstitial tissue may reflect the migration of these cells and their participation in the pathological process of RMS. Recent studies indicated that after early inflammation in ARF, the presence of inflammatory cells and increased expression of several cytokines in the heart valve tissues remains a subclinical inflammatory response that ultimately leads to permanent valve injury (1). As an anti-inflammatory cytokine, IL-35 may have a role either locally in fibrogenesis/calcification of heart valves as the sequelae of RHD or systemically in regulating overt inflammatory responses. Our finding of high levels of IL-35 in the blood and MV tissues of patients with advanced RMS but no evidence of systemic inflammation supports such a notion, although the underlying mechanisms remain to be investigated.

Hu et al. reported that IL-35 pretreatment reduced lipopolysaccharide (LPS) -induced inflammatory responses, apoptosis, and fibrotic reactions in the myocardium of mice, suggesting IL-35 as a promising therapeutic target for sepsis-induced cardiac injury (29). Further research revealed that IL-35 alleviated LPS-induced endothelial dysfunction by inhibiting endothelial-to-mesenchymal transition in human umbilical vein endothelial cells and in the thoracic and abdominal aortas of mice (30). It is well established that T effector cells play a crucial role in the cross recognition between streptococcal antigens and valvular proteins, contributing to inflammation and autoimmunity in heart valves. Given IL-35’s significant physiological functions, such as suppressing Th1 and Th17 cell proliferation and facilitating the conversion of Th2 cells into regulatory T cells (31), we propose that IL-35 holds the potential as a targeted therapy for RMS.

However, further research is needed to uncover the underlying molecular mechanisms, including the origin IL-35 and its role in preventing valve damage in RMS. Identifying the specific cells that secrete IL-35 in RMS is crucial for further exploring its immune mechanisms. Some research show that Tregs secrete IL-35, which in turn promotes their function, playing an critical role in immune regulation and maintaining immune balance. By influencing Tregs development and exerting immunosuppressive effects, IL-35 is particularly significant in cancer immunology (32). Tregs are essential for preserving immunological homeostasis, suppressing inflammation, and ensuring self-tolerance. IL-35 has been investigated as a potential strategy for enhancing immune responses against malignancies, with major clinical trials exploring Treg/IL-35-based therapies in lung, breast, and colorectal cancers (33). Some research suggests that IL-35 produced by Bregs plays a role in certain autoimmune diseases, such as autoimmune uveitis and multiple sclerosis (34, 35). IL-35+ Bregs contribute to immune tolerance in chronic infections by supporting both IL-35-producing Tregs and their own expansion (16). This suggests a functional interaction between IL-35+Bregs and IL-35+Tregs. Therefore, we speculate that both Bregs and Tregs may produce IL-35 in RMS, which requires further in-depth research for validation.

To advance our research, a significant expansion of the sample size is necessary to determine whether IL-35 can be used as a biomarker of valve damage in RMS. Further studies are essential to deepen our understanding of IL-35 biology and its therapeutic potential for RMS.

## 6 Conclusion

This is the first report of an association between IL-35 and RMS. We found increased IL-35 expressions in RMS patients’ blood and MV tissues. The level of IL-35 expression correlates with the severity of RMS. These results suggest that IL-35 may play an important role in the development of RMS.

## Data Availability

The original contributions presented in the study are included in the article/supplementary material. Further inquiries can be directed to the corresponding author.
